# Fulfilling the criteria for CLP classification: the implications for substances under the EU chemicals legislation

**DOI:** 10.3389/ftox.2024.1452065

**Published:** 2024-10-03

**Authors:** Diana Kättström, Anna Beronius, Urban Boije af Gennäs, Christina Rudén, Marlene Ågerstrand

**Affiliations:** ^1^ Department of Environmental Science, Stockholm University, Stockholm, Sweden; ^2^ Institute of Environmental Medicine, Karolinska Institutet, Stockholm, Sweden; ^3^ Swedish Chemicals Agency, Sundbyberg, Sweden

**Keywords:** CLP, hazard classification, hazard criteria, EU legislation, chemical strategy for sustainability

## Abstract

The CLP mandates manufacturers and importers to classify substances and mixtures according to hazard criteria, with notifications submitted to the European Chemicals Agency (ECHA). Substances meeting hazard criteria must be appropriately labelled and packaged to communicate hazards effectively. The CLP establishes hazard classification criteria but does not independently prohibit or restrict the use of hazardous chemicals. Instead, it serves as a basis for regulatory obligations in other specific regulations. This study investigates the regulatory implications of meeting hazard criteria under the CLP across EU regulations and directives listed in EU Chemicals Legislation Finder (EUCLEF). The results show that fulfilling criteria for human health hazard classes trigger regulatory obligations in the highest number of regulations/directives, with carcinogenicity, mutagenicity, and reproductive toxicity (CMR) leading to obligations in 19 of 20 pieces of legislation linked to the CLP. Conversely, physical, environmental, and ozone layer hazards are associated with fewer regulations and directives, and lead to fewer prohibitions. The study underscores the pivotal role of the CLP in EU chemical legislation and the need for coherence and consistency across regulations. While regulatory obligations are primarily aimed at substances meeting hazard criteria, the variability in self-classification notifications and limitations in harmonized classification processes were observed. Moreover, the complexity of the regulatory structure poses challenges for stakeholders and policymakers, including inconsistencies, compliance difficulties, and the need for frequent revisions. Addressing these challenges is critical for enhancing regulatory effectiveness and ensuring a more coherent and harmonized approach to chemical management in the EU.

## Introduction

The Globally Harmonized System of Classification and Labelling of Chemicals (GHS) is an international non-binding agreement developed by the United Nations. The primary objective of the GHS is to enhance the protection of human health and the environment from hazardous chemicals by establishing a harmonized system for hazard classification and information. It also aims to provide a foundation for national chemical programs in countries without an existing system (United Nations, 2021). In the European Union (EU), the GHS is implemented through Regulation (EC) 1272/2008 on the classification, labelling, and packaging of substances and mixtures (CLP), which has repealed Directives 67/548/EEC and 1999/45/EC.

In essence, the CLP mandates the manufacturers and importers of substances and mixtures to classify them according to the hazard criteria established by the regulation, based on the available data. The classification should be notified to the European Chemicals Agency (ECHA), which maintains an inventory of all classification notifications. Substances and mixtures fulfilling the criteria for any of the hazards need to be properly labelled and packaged to enable the communication of hazards (European Parliament and Council of the European Union, 2008).

A substance can either be classified via the process of self-classification, or through harmonised classification. Self-classification means that a manufacturer or an importer of a substance or mixture makes the assessment. Substances meeting the criteria for respiratory sensitisation, germ cell mutagenicity, carcinogenicity and reproductive toxicity may be subject to harmonised classification, while active substances used in plant protection products or biocidal products must be subject to harmonised classification for all hazard classes. Harmonised classification for active substances used in plant protection products and biocidal products is initiated by a competent authority of a Member State, whereas under other legislation it may in some cases be initiated by a manufacturer, importer or downstream user. A proposal for harmonised classification is submitted to ECHA, where a the Committee for Risk Assessment (RAC) adopts an opinion and forwards it to the European Commission for decision (European Parliament and Council of the European Union, 2008).

The CLP provides criteria for classification of 17 physical, 11 human health, and four environmental hazards, as well as one hazard class for the ozone layer. Four hazard classes were recently added to the CLP: endocrine disruption for human health (ED HH), endocrine disruption for the environment (ED ENV), persistent, bioaccumulative, and toxic (PBT) and very persistent and very bioaccumulative (/vPvB), and persistent, mobile, and toxic (PMT) and very persistent and very mobile (vPvM). However, the introduction of these new hazard classes into the GHS is still under discussion (European Parliament and Council of the European Union, 2008). The changes in the CLP are a result of the Chemicals Strategy for Sustainability (European Commission, 2020), launched by the European Commission in 2020. The Strategy is a long-term vision for sustainable management of chemicals in the EU and aims at enhancing the protection of human health and the environment from harmful chemicals and moving towards a toxic-free environment (European Commission, 2020). The actions proposed in the Strategy aim to further strengthen the CLP as the centrepiece of EU chemicals legislation by introducing the so-called generic approach to risk management, which allows chemicals to be managed on the basis of hazard (European Commission, 2020). In addition, the CLP will play an important role in achieving the aim of “one substance, one assessment,” a key deliverable of the Chemicals Strategy for Sustainability. Some of the proposals within “one substance, one assessment” are: a common data platform, enhanced coordination of regulatory processes between different pieces of legislation and regulatory agencies, and strengthened detection and faster action on hazardous substances and mixtures.

Meanwhile, the CLP does not, on its own, prohibit or restrict the use of hazardous chemicals, nor does it contain any data requirements. Instead, it sets a framework for the classification of chemicals based on available data. Such hazard classification may then serve as a basis for regulatory obligations in other chemicals regulations. For example, while the CLP provides criteria for the classification of substances as carcinogenic, it does not require testing for carcinogenic properties, nor does it limit the use of carcinogenic substances. Rather, the use of substances classified as carcinogenic is prohibited or restricted in various products and articles by other regulations, such as the Toy Safety Directive or the Cosmetic Products Regulation. In addition, the hazard information communicated through warning labelling might lead to risk reduction if the use of classified substances or products are used with appropriate safety measures to reduces exposures, or the use volumes reduced or by substitution processes where hazardous chemicals and products are replaced by more benign alternatives.

The interface between the CLP and other related chemicals regulations was analysed during a fitness check under the Regulatory Fitness and Performance Programme (REFIT) (European Commission Directorate-General for Internal Market I et al., 2017). However, the main focus of the fitness check was to provide a summary of the risk management measures under selected pieces of chemicals legislation to support the evaluation of the efficacy, efficiency, relevance, coherence and added EU value. This paper aims to further investigate the linkage between the CLP, and the relevant regulations and directives, by mapping out all regulatory obligations triggered when a chemical fulfills the criteria for a hazard class. Furthermore, a case study was conducted to compare the regulatory implications of classifying a substance as carcinogenic and toxic to the aquatic environment. Understanding the connections between CLP and risk management measures in other regulations could help address regulatory inconsistencies and facilitate better harmonization across the EU chemicals legislation.

## Methods

The relevant EU chemicals regulations were identified using the EU Chemicals Legislation Finder (EUCLEF) available on the website of ECHA. The latest consolidated versions of the identified legislation were accessed through the website Access to European Union Law (EUR-Lex) run by the Publications Office of the European Union. The documents were screened for relevance to the CLP using the following search terms: “1272/2008,” “hazardous,” and “classif.” Legislations that did not contain the above search words were excluded.

In this paper, the term “regulatory obligations” is used to denote mandatory regulatory requirements, applicable to substances and/or mixtures, for manufacturers, importers or other organizations, to complete or refrain from, in order to be compliant with the applicable law. Regulatory obligations for substances or mixtures meeting the criteria or classified under the CLP were extracted from each relevant piece of legislation and compiled in a Microsoft Excel database. The name and number of the article containing the regulatory obligation, as well as the CLP hazard class to which it applies were also collected. In addition, information on whether the regulatory obligation was based on self-classification or harmonised classification, and if it applied generally to all CLP hazard classes or specific ones was also collected. Subsequently, each obligation was assigned a category linked to the risk reduction potential of the regulatory obligation. The categories and their descriptions are presented in Table 1.

**TABLE 1:** Categories assigned to regulatory obligations and their description.

Category	Description
Prohibition	The use of the substance is prohibited
Conditioned use	The use of the substance is restricted or specific conditions apply
Exemption from a simplified procedure	The substance may not follow the simplified procedure of approval, assessment or registration
Prioritization	The substance is prioritized for risk management measures
Assessment	Additional assessment is required for the substance
Other preventive/risk mitigation measures	Other measures aiming at preventing emissions or at removal or control of the contamination by the substance
Information requirement	Additional information is required for the substance or should be made available to professional users, consumers or Member State Competent Authorities

A case study was conducted to illustrate the types of and the differences in regulatory obligations that exist under the EU chemicals legislation for substances that meet different CLP hazard criteria. The case study exemplifies the regulatory obligations triggered by classification as “hazardous to the aquatic environment” (Aquatic Acute/Chronic) and “carcinogenicity” (Carc.) The hazard class “Hazardous to the aquatic environment” was the only environmental hazard class in use at the time of data collection and analysis, while “Carcinogenic” was chosen to illustrate the maximum regulatory obligations.

## Results

EUCLEF listed 59 regulations and directives on chemicals referring to the selected keywords. Out of these, six are no longer in force, and 33 do not trigger any regulatory obligations based on CLP classifications. In total, 20 regulations contain regulatory obligations for substances and/or mixtures classified according to the CLP, as shown in Table 2. The full data file with regulations, articles and article names, as well as related regulatory obligations, is available in Supplementary Materials.

**TABLE 2:** Overview of the regulations and directives containing regulatory obligations for substances classified as having physical, human health, environmental hazards or hazards to the ozone layer. The document number of the examined version of each piece of legislation is given below the name.

	Physical hazards	Human health hazards	Environmental hazards	Hazard to the ozone layer
Aerosol Dispenser Directive, 75/324/EEC 01975L0324-20180212	yes	no	no	no
Biocidal Products Regulation, (EU) 528/2012 02012R0528-20220415	yes	yes	yes	yes
Chemical Agents Directive, 98/24/EC 01998L0024-20190726	yes	yes	no	no
Carcinogens and Mutagens Directive, 2004/37/EC 02004L0037-20220405	no	yes	no	no
Cosmetic Products Regulation, (EC) 1223/2009 02009R1223-20230816	yes	yes	yes	yes
EU Ecolabel Regulation, (EC) 66/2010 02010R0066-20171114	no	yes	yes	no
End-of-Life Vehicles Directive, 2000/53/EC 02000L0053-20200306	yes	yes	yes	yes
Food Contact Active and Intelligent Materials and Articles Regulation, (EC) 450/2009 32009R0450	no	yes	no	no
Industrial Emissions Directive, 2010/75/EU 02010L0075-20110106	yes	yes	yes	yes
*In Vitro* Diagnostic Medical Devices Regulation, (EU) 2017/746 02017R0746-20230320	no	yes	no	no
Medical Devices Regulation, (EU) 2017/745 02017R0745-20230320	no	yes	no	no
Pressure Equipment Directive, 2014/68/EU 02014L0068-20140717	yes	yes	no	no
Prior Informed Consent Regulation, (EU) 649/2012 02012R0649-20220701	no	yes	no	no
Plastic Materials and Articles Regulation, (EU) 10/2011 02011R0010-20230831	no	yes	no	no
Protection of Pregnant and Breastfeeding Workers Directive, 92/85/EEC 01992L0085-20190726	no	yes	no	no
Plant Protection Products Regulation, (EC) 1107/2009 02009R1107-20221121	yes	yes	yes	yes
Protection of Young People Directive, 94/33/EC 01994L0033-20190726	yes	yes	no	no
Registration, Evaluation, Authorisation and Restriction of Chemicals Regulation, (EC) 1907/2006 (REACH) 02006R1907-20230629	yes	yes	yes	yes
Toy Safety Directive, 2009/48/EC 02009L0048-20221205	no	yes	no	no
Waste Framework Directive, 2008/98/EC 02008L0098-20180705	yes	yes	yes	yes

In general, the regulatory obligations refer to the CLP criteria in three ways, using either “substances that fulfil/meet the criteria,” “substances classified as,” or “substances classified as … under/in accordance with Part 3 of Annex VI,” which specifically refers to the harmonised classification under CLP. Although not explicitly stated, “substances classified as” may refer to substances with harmonised or self-classification, the classification by a notifier may not be required for “substances that fulfil/meet the criteria.” However, in the context of this paper, the two will be used interchangeably as both imply that the substance has the properties specified in the criteria of the CLP hazard class.

Six regulations/directives contain at least one regulatory obligation applicable to all CLP hazard classes i.e., physical, human health, environmental and hazards to the ozone layer. Eight regulations/directives only contain regulatory obligations for substances and/or mixtures classified as hazardous to human health. In total, seven regulations/directives prohibit substances and/or mixtures fulfilling certain CLP hazard criteria: Biocidal Products Regulation, Cosmetic Products Regulation, EU Ecolabel Regulation, Prior Informed Consent Regulation, Plant Protection Products Regulation, Protection of Young People Directive, and Toy Safety Directive.

The majority of regulatory obligations applied to substances when the CLP classification criteria were fulfilled. Only four regulations imposed obligations specifically triggered by harmonized classification under the CLP, see Supplementary Materials. These contained restrictions under REACH, the prohibition of the use of CMR substances under the Cosmetic Products Regulation, and the requirement to minimize the risks from hazardous substances in medical devices and *in vitro* medical devices under the Medical Devices Regulation and the *In Vitro* Diagnostic Medical Devices Regulation.

**FIGURE 1:**
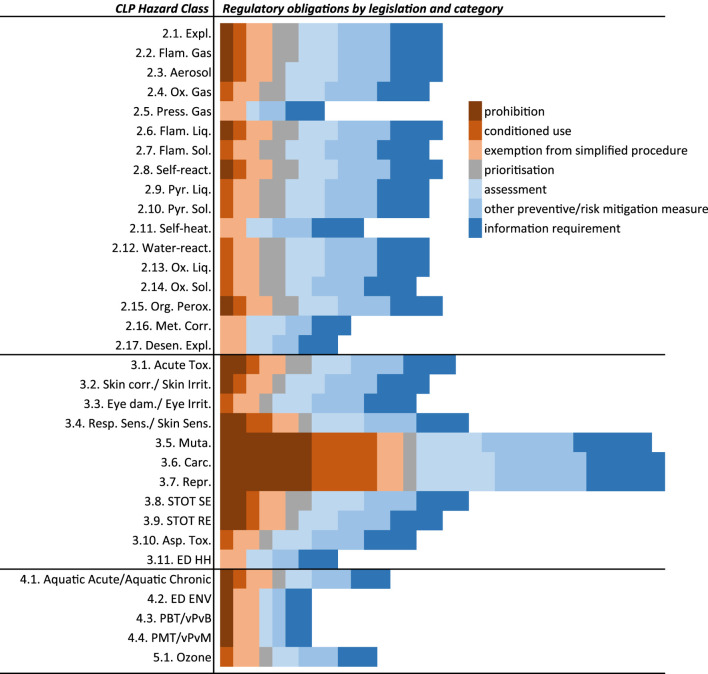
Visualisation of regulatory obligations for substances classified as hazardous under CLP. Each cell represents a regulatory obligation under a regulation or directive. The regulatory obligations are grouped into the following categories as described in Table 1: prohibition, conditioned use, exemption from simplified procedure, prioritisation, assessment, other preventive/risk mitigation measure, and information requirement.

Figure 1 shows existing regulatory obligations for each regulation/directive per category and hazard class. On the whole, fulfilling criteria for human health hazards triggered regulatory obligations in the highest number of regulations/directives followed by classifications for physical, environmental, and hazards for the ozone layer.

Among the human health hazards, carcinogenicity, mutagenicity, and reproductive toxicity (CMR) were linked to the highest number of regulations/directives. Fulfilling the criteria for CMR triggered regulatory obligations in 19 regulations/directives, and led to the highest number of prohibitions, with seven regulations/directives banning the use of, or exposure to, such substances and/or mixtures.

Eleven pieces of legislation were linked to at least one physical hazard class. Fulfilling the criteria for explosives, flammable gases and liquids, self-reacting substances and mixtures, and organic peroxides triggers regulatory obligations in ten regulations/directives. Together with the hazard class for aerosols, these were the only physical hazard classes for which prohibition existed. The Protection of Young People Directive prohibits the employment of young people for work involving harmful exposure to such substances and/or mixtures. Physical hazard classes with links to the least number of regulations/directives were gases under pressure, corrosive to metals, and desensitised explosives. The hazard class for the ozone layer was linked to seven regulations/directives. The environmental hazard class, for substances and/or mixtures hazardous to the aquatic environment, was linked to eight different regulations/directives. As expected, the new environmental hazard classes i.e., endocrine disruptive for the environment, PBT/vPvB, and PMT/vPvM, triggered the least number of regulatory obligations. For substances or mixtures meeting the criteria for any of the environmental hazard classes, prohibition existed only in one regulation, the EU Ecolabel Regulation, which prohibits awarding the EU Ecolabel to goods that contain substances, or to mixtures, meeting the criteria.

In total, nine regulations/directives had definitions based on CLP hazard classes: Biocidal Products Regulation, Chemical Agents Directive, Carcinogens and Mutagens Directive, End-of-Life Vehicles Directive, Industrial Emissions Directive, Plant Protection Products Regulation, REACH, Water Framework Directive, and Waste Framework Directive. For example, the Biocidal Products Regulation defined “substance of concern,” “candidate for substitution,” and “active substances give rise to concern” directly based on CLP hazard classes. Plant Protection Products Regulation linked “substance of concern,” “basic substances,” “candidate for substitution,” and “substance of low-risk” to CLP hazard classes. In addition, the PBT criteria in REACH and Plant Protection Products Regulation considered the criterion for toxicity (T) to be fulfilled if meeting CLP hazard criteria for CMR or specific target organ toxicity-repeated exposure (STOT RE).

### Case study: fulfilling criteria for hazardous to aquatic environment vs. carcinogenicity

#### Fulfilling criteria for hazardous to the aquatic environment

For substances meeting the criteria of being hazardous to the aquatic environment, regulatory obligations were found in eight regulations and directives. Under **REACH**, a supplier of a substance or mixture hazardous to the aquatic environment is required to provide the recipient with a safety data sheet, containing information about the properties and hazards of the substances/mixture and instructions for handling and disposal [Article 31 (1) (a)]. For substances for which chemical safety assessment is performed, exposure assessment, including exposure scenarios, and risk characterization needs to be included [Article 14 (4)]. ECHA is required to prioritize the testing proposals for such substances and mixtures, and make information about their properties and classification publicly available [Article 40 (1)]. A liquid substance or mixture meeting the criteria for being hazardous to the aquatic environment is restricted from being used in some specific articles, such as articles for tricks and jokes, or games for more than one participant (Annex XVII entry 3). If substances hazardous to the aquatic environment were to be used as active substances in plant protection products under the **Plant Protection Products Regulation** [Article 47 (1)], or biocidal products under the **Biocidal Products Regulation** (Article 25), they are defined as substances of concern and products containing such substances may not be authorized following the simplified procedure, which allows the product to be placed on the whole EU market without the need of mutual recognition from the Member States. Under the **EU Ecolabel Regulation**, an ecolabel may neither be awarded to articles that contain substances hazardous to the aquatic environment, nor mixtures meeting those criteria [Article 6 (6)]. The **Cosmetic Products Regulation** requires quantitative information about the composition to be made available for hazardous substances (Article 21). The **Waste Framework Directive** requires the Member States to ensure that the hazardous substances, mixtures and components are removed from the hazardous waste [Article 10 (5)], and that human health and the environment are protected during production, collection, transport and storage of the hazardous waste (Article 17). In addition, the Member States shall ensure that the hazardous waste is packaged and labelled during collection, transport and temporary storage, and accompanied by an identification document [Article 19 (1-2)]. The Member States should also ensure that the waste management plans include information on any special arrangement for hazardous waste [Article 28 (3)]. The establishments dealing with hazardous waste shall be regularly inspected by the competent authorities [Articles 34 (1)], and keep records of quantities, nature and origin of the hazardous waste [Article 35 (1-2) (4)]. The **End-of-life Vehicle Directive** states that Member States should encourage vehicle manufacturers to limit the use of hazardous substances [Article 4 (1)], and take measures to ensure that the producers provide information on the location of the hazardous substances in the vehicle [Article 8 (3)]. For industrial sites, the Industrial Emissions Directive mandates Member States to ensure that permits contain measures for regular maintenance, surveillance, and monitoring of soil and groundwater to prevent contamination [Article 14 (1)]. The directive also requires the operator of the site to prepare and submit a report on the presence of the relevant hazardous substances, assess the state of the soil and groundwater upon the closure of the site, and take actions to remove or reduce the contamination of the site once the activities have ceased (Article 22). It is important to note that all of the above-mentioned regulatory obligations also apply to all other hazard classes, including carcinogenicity.

#### Fulfilling criteria for carcinogenicity

For a substance meeting the hazard criteria for carcinogenicity, additional regulatory obligations across 11 regulations and directives apply. Substances meeting the criteria for carcinogenicity may be included in Annex XIV of **REACH**, which lists substances for which restrictions exist (Article 57). **REACH** requires that for substances that meet the criteria for carcinogenicity, the name in the IUPAC nomenclature must be made publicly available on the internet free of charge [Article 119 (1)]. With some exceptions, the use and placing on the market of substances with harmonised classification as carcinogen category 1A or 1B is restricted for supply to the general public (Annex XVII entry 28). In addition, the placing on the market of carcinogens category 1A, 1B or 2 with harmonised classification is restricted in tattoo ink (Annex XVII entry 75). Annex XVII of **REACH** also places specific concentration limits on Polycyclic-aromatic Hydrocarbons (PAH) that have harmonised classification as carcinogenic category 1 or 2 (Annex XVII, Appendix 13, entry 75). Under the **Biocidal Products Regulation** and **Plant Protection Products Regulation**, substances meeting the criteria for carcinogenicity 1A or 1B shall generally not be approved as active substances [Article 5 (1) of the Biocidal Products Regulation, Annex II, 3.6.3 of the Plant Protection Products Regulation]. However, under certain circumstances and for a limited time, they may be approved as candidates for substitution [Article 10 (4) of the Biocidal Products Regulation, Article 24 (1) of the Plant Protection Products Regulation]. For such substances, the **Biocidal Products Regulation** limits the approval to a maximum of 5 years [Article 4 (1)] and the **Plant Protection Products Regulation** to a maximum of 7 years [Article 24 (1)]. Biocidal products containing candidates for substitution may not be authorized for use by the general public [Article 19 (4)]. The authorization process for these biocidal products requires a comparative assessment, and if specific conditions are met, the product may be authorized for up to 5 years (Article 23). Similarly, the **Plant Protection Products Regulation** may authorise plant protection products with candidates for substitution for a limited time only, and only after a comparative assessment has been performed [Articles 4 (7) and 50 (1)]. Under the **Prior Informed Consent Regulation**, substances for which the PIC procedure with prior notification and approval applies, may not be exported if they are classified as carcinogenic category 1A or 1B [Article 14 (7)]. For industrial sites, the **Industrial Emissions Directive** requires the substances classified as carcinogens to be replaced by less harmful substances as soon as possible (Article 58). To protect the workers, the Carcinogens and Mutagens Directive puts obligations on the employer to perform an assessment of the exposure to carcinogens, reduce the use of such substances, prevent the risks from the exposure and take other necessary precautionary measures [Articles 3(2), 4(1), 5]. In addition, the health of workers should be monitored, their medical records preserved for at least 40 years, and the Competent Authority should be notified of any cases of cancer that occur after exposure to carcinogens [Articles 6, 10 (1), 11, 14–16, 18]. Likewise, the **Chemical Agents Directive** aims at protecting the health of workers by requiring the employer to assess if any hazardous substances are present in the workplace and if so, assess the risks to the health of workers and put in place preventive measures [Articles 3(1), 4(1)]. The employer shall make available information about the hazardous substances, such as safety data sheets, and about any precautionary measure or emergency procedure (Articles 7, 8, 10). The **Protection of Young People Directive** prohibits the employment of young workers under the age of 18 for work that involves substances meeting the criteria of carcinogenicity of any category [Annex, entry 3(a)]. The **Protection of Pregnant and Breastfeeding Workers Directive** mandates the employer to assess the exposure of pregnant workers, and workers who have recently given birth or are breastfeeding to carcinogens of any category and to take necessary measures to mitigate the risks [Annex I, entry 3(a)]. According to the **Food Contact Active and Intelligent Materials and Articles Regulation**, carcinogenic substances may not be used in components of materials that aim to extend the shelf-life or monitor the condition of food even if they are not in direct contact with food [Article 5(c)]. The **Plastic Materials and Articles Regulation** restricts the use of carcinogens in plastic multi-layer materials and multi-material multi-layer materials, even if the carcinogens do not come into direct contact with food (Articles 13, 14). Substances with harmonised classification as carcinogens category 1A or 1B are prohibited by the **Cosmetic Products Regulation** from the use in cosmetic products. Carcinogens of category 2 with harmonised classification are also prohibited but may be used if evaluated and found safe by the expert committee (Article 15). The ***In Vitro* Diagnostic Medical Devices Regulation** and the **Medical Devices Regulation** mandate that the *in vitro* medical devices and medical devices should be designed and manufactured so that the level of substances with harmonised classification as carcinogens of any category would be as low as possible (Annex I, entry 10). The **Medical Devices Regulation** also requires that the information about precautions related to presence of the carcinogens in the medical device should be included in the instructions provided to the user/patient (Annex I, chapters II and III). The **Toy Safety Directive** prohibits the use of substances classified as carcinogens of any category to be present in toys or parts or components of toys (Annex II, chapter III, entry 3).

## Discussion

This paper aimed to examine what regulatory obligations exist for substances and/or mixtures that meet the CLP hazard criteria and to illustrate the differences that may exist between obligations for different hazards. Our results showed that the CLP was linked to 20 of the 53 regulations or directives listed in EUCLEF. Meeting the criteria for human health hazard classes triggered regulatory obligations in the largest number of regulations/directives. In particular, meeting the criteria for CMRs led to regulatory obligations in 19 pieces of legislation, with bans on the use of or exposure to such substances in seven of them. In contrast, physical, environmental and hazards to the ozone layer, were linked to fewer regulations and directives, and triggered fewer prohibitions. The majority of the regulatory obligations were aimed at substances meeting the hazard criteria, with few specifically aimed at those with harmonised classifications.

During the analysis, some ambiguities were revealed. One such ambiguity is found in the EU Ecolabel Regulation. Article 6 (6) states that the Ecolabel may not be awarded to substances that are “toxic, hazardous to the environment, carcinogenic, mutagenic or toxic to reproduction, in accordance with Regulation (EC) 1272/2008.” However, it is not clear which hazard classes are meant by “toxic.” This may refer to either or all of, the following hazard classes: acute toxicity, specific target organ toxicity single exposure or specific target organ toxicity repeated exposure. Another example of ambiguity is found in Construction Products Regulation where it refers to both “hazardous substances” [article 67 (1)] and “dangerous substances” in part 3 of Annex I without referring to either the CLP, its predecessors Directives 67/548/EEC and 1999/45/EC or in other way defining these terms. Similarly, WEEE refers to hazardous substances in articles 3, 6, 14, part 3 of Annex VI without mentioning the CLP or defining “hazardous.”

For this paper, the terms “meets the criteria” and “classified” have been used interchangeably. The reasoning behind this was that if a substance is determined to meet the hazard criteria, it should be classified by. However, the two may potentially refer to two different groups of substances. If “classified” means that the substance meets the criteria and a hazard classification is reported to the Classification and Labelling Inventory by the manufacturer or downstream user, then “meets the criteria” could refer to the broader list of substances. Such ambiguity, although possibly not significant for the outcome, may create unnecessary confusion for the industry, and potentially lead to different application of the regulatory obligations that exist for substances with hazardous properties.

As evident from our results, a classification as hazardous to human health under the CLP appears to be more likely to result in downstream measures for risk reduction compared to a classification as hazardous to the environment. This finding was not surprising. Many of the linked pieces of legislation applied to substances and mixtures present in products and articles for use by consumers, workers and professionals, thus focusing on the protection of human health. Restricting the use of substances that are hazardous to human health can also reduce environmental exposure to these substances. However, strengthening of the environmental protection could also be motivated. Recent research points to anthropogenic chemical pollution as a driver for adverse effects on the environment globally, including the loss of biodiversity (Bernhardt et al., 2017; Sigmund et al., 2023).

As described, a classification as hazardous to the environment under the CLP Regulation is not linked to as many downstream actions as a human health classification. Nevertheless, several regulations include regulatory obligations for substances with PBT/vPvB properties. Currently, these regulatory obligations are based on criteria contained in REACH and the Plant Protection Products Regulation. However, as the PBT/vPvB hazard class recently were introduced to the CLP, a revision is needed to link future regulatory obligations to the new CLP criteria. Furthermore, the addition of new hazard classes for environmental endocrine disrupters and PMT/vPvM to the CLP could, if linked to downstream regulatory obligations, further improve environmental protection.

As previously mentioned, under the CLP, a substance can either be classified by a process of harmonised classification or by self-classification. There are advantages and disadvantages with both systems, but they remain operational next to each other. In some regulations, the regulatory obligations were reserved for substances with a harmonised classification. One example was the Cosmetic Products Regulation which prohibited the use of CMR substances with a harmonised classification. The assessment of properties for harmonised classification is performed by a Member State competent authority, reviewed by ECHA and, finally, decided by the European Commission. Such a harmonised entry can reduce variability in classifications and improve the quality and reliability of the assessment, but it is a time-consuming process that limits the number of substances that can be classified. In 2020, harmonised classifications were adopted for 48 substances, with each one requiring 6-7 years for the completion of the process (European Chemicals Agency, 2021). Thus, having regulatory obligations for substances with harmonised classifications might be less protective given that only a subset of substances with hazardous properties will receive such classification.

The majority of the regulatory obligations, however, concern substances classified by self-classification. As discussed above, this approach may be more protective, but it is not without issues. A well-known problem with self-classification is that the data used to determine whether or not a substance meets the criteria can vary from company to company. This is evidenced by the differences in classification reported by different companies (European Commission Directorate-General for Internal Market I et al., 2017; European Chemicals Agency, 2021; European Commission, 2019). Hypothetically, if individual companies arrive at different conclusions about whether a substance meets the criteria for a hazard class, they may also invoke downstream regulatory obligations accordingly. This issue could be partially addressed by establishing a common open data platform for sharing and re-using data and facilitating access to relevant data, as envisaged by the Chemicals Strategy for Sustainability (European Commission, 2020).

The overall structure of EU chemicals legislation consists of REACH and CLP, complemented by a large number of regulations and directives regulating specific uses and products. The EU Chemicals Legislation Finder, EUCLEF, listed over fifty functional regulations and directives, each with its specific design, objective and regulatory obligations. During the data collection and analysis, we found a variation in the design of the regulations and directives. Some based the regulatory obligations on the intrinsic properties of the substances and referred to the CLP hazard classes, others listed specific substances to which the obligations applied, while a few required assessment and authorisation of individual substances prior to marketing. This structure presents several challenges for the chemical industry, downstream users, the Member State competent authorities, as well as for the EU policymakers. First of all, such a structure is prone to inconsistencies between the regulations, such as gaps or overlaps. Our previous research showed that the regulation of antimicrobial substances used in biocidal products under the Biocidal Products Regulation was stricter compared to when the same substances were used in cosmetic products under the CPR (Kättström et al., 2022). Further, the complexity of the structure might create difficulties for the industry to identify relevant legislation and comply with the requirements. Finally, revisions of several regulations may be required when amending individual pieces of legislation. For example, the impact of the recent introduction of the new hazard classes under the CLP may be limited due to the differences in how individual regulations and directives refer to the CLP. In regulations and directives, where regulatory obligations are general and apply to substances classified in any hazard class under the CLP, the new hazard classes will automatically be implemented. In contrast, regulations and directives that refer to specific CLP hazard classes will require revision for the new hazard classes to be introduced.

More than half of the chemicals regulations examined in this study did not use the CLP criteria for identifying or managing the risks of hazardous chemicals. They also did not refer to Directives 67/548/EEC and 1999/45/EC, which preceded the CLP. Some examples of regulations not connected to the CLP are the Detergents Regulation (EC) 648/2004, the Restriction of Hazardous Substances in Electrical and Electronic Equipment Directive 2011/65/EU, and the Water Framework Directive 2000/60/EC. To determine whether or not these regulations could be linked to the CLP and whether or not this would lead to better regulation of hazardous substances, a thorough examination of each regulation, its scope, objective and processes would be required. Such an evaluation would be in line with the Chemicals Strategy for Sustainability.

## Data Availability

The original contributions presented in the study are included in the article/Supplementary Material, further inquiries can be directed to the corresponding author.
